# Hip resurfacing arthroplasty: short-term survivorship of 4,401 hips from the Finnish Arthroplasty Register

**DOI:** 10.3109/17453674.2012.693016

**Published:** 2012-06-04

**Authors:** Matti Seppänen, Keijo Mäkelä, Petri Virolainen, Ville Remes, Pekka Pulkkinen, Antti Eskelinen

**Affiliations:** ^1^Department of Orthopaedics and Traumatology, Turku University Hospital, Turku; ^2^Department of Orthopaedics and Traumatology, Helsinki University Central Hospital, Helsinki; ^3^Department of Public Health, Helsinki University, Helsinki; ^4^Coxa Hospital for Joint Replacement, Tampere, Finland.; Correspondence: keijo.makela@tyks.fi

## Abstract

**Background and purpose:**

Population-based registry data from the Nordic Arthroplasty Register Association (NARA) and from the National Joint Register of England and Wales have revealed that the outcome after hip resurfacing arthroplasty (HRA) is inferior to that of conventional total hip arthroplasty (THA). We analyzed the short-term survival of 4,401 HRAs in the Finnish Arthroplasty Register.

**Methods:**

We compared the revision risk of the 4,401 HRAs from the Register to that of 48,409 THAs performed during the same time period. The median follow-up time was 3.5 (0–9) years for HRAs and 3.9 (0–9) years for THAs.

**Results:**

There was no statistically significant difference in revision risk between HRAs and THAs (RR = 0.93, 95% CI: 0.78–1.10). Female patients had about double the revision risk of male patients (RR = 2.0, CI: 1.4–2.7). Hospitals that had performed 100 or more HRA procedures had a lower revision risk than those with less than 100 HRAs (RR = 0.6, CI: 0.4–0.9). Articular Surface Replacement (ASR, DePuy) had inferior outcome with higher revision risk than the Birmingham Hip Resurfacing implant (BHR, Smith & Nephew), the reference implant (RR = 1.8, CI: 1.2–2.7).

**Interpretation:**

We found that HRA had comparable short-term survivorship to THA at a nationwide level. Implant design had an influence on revision rates. ASR had higher revision risk. Low hospital procedure volume worsened the outcome of HRA. Female patients had twice the revision risk of male patients.

Good short-term results of using modern hip resurfacing devices have been reported from pioneering centers (Amstutz et al. 2004, Daniel et al. 2004). Recently, these results have been confirmed by independent studies (Hing et al. 2007a, Heilpern et al. 2008, Steffen et al. 2008, Khan et al. 2009). However, there have been a variety of early complications of HRA, such as femoral neck fracture, aseptic loosening of the femoral component, and metallosis of the hip joint with soft-tissue necrosis (Shimmin et al. 2005, Keegan et al. 2007, Grammatopolous et al. 2009, Ollivere et al. 2009). Registry data have revealed that the early revision rate of HRA is higher than that of THA (Australian Orthopaedic Association, Johanson et al. 2010). Furthermore, conventional stems can nowadays be used with a large metal-on-metal (MoM) articulation similar to that in HRA. We examined the early outcome of HRA and compared it to that of THA using data in the Finnish Arthroplasty Register.

## Patients and methods

### The Finnish Arthroplasty Register

Since 1980, the Finnish Arthroplasty Register has been collecting information on total hip replacements (Paavolainen et al. 1991). Healthcare authorities, institutions, and orthopedic units are obliged to provide the National Institute for Health and Welfare with information essential for maintenance of the register. Since 1995, the data in the register have been compared with those of hospital discharge registries at regular intervals. Currently, 98% of implantations are recorded.

### Study population and inclusion criteria

During the study period (2001–2009), 48,409 primary THRs and 4,401 primary HRAs were performed in Finland for primary or secondary osteoarthritis. To reduce the skew in the demographic distribution between patients operated with HRA and those operated with THA, patients older than 85 years of age were excluded (the oldest patient operated with HRA was 85 years old). Also, those patients with another diagnosis (including fractures and avascular necrosis of the femoral head) or rheumatoid arthritis were excluded. Only HRA designs used in more than 90 operations during the study period and with more than 20 hips at risk at 5 years were included. These criteria permitted the inclusion of 6 HRA designs.

### Statistics

Kaplan-Meier survival analysis was used to calculate the survival probabilities of implants with 95% confidence interval (95% CI). These survival data were compared using the log-rank test. Patients who died or left Finland during the follow-up period were censored at that point. Adjusted revision rates were calculated using Cox multiple regression analysis. The proportional hazards assumption was controlled for by visual inspection of the Cox curves. Inclusion of bilateral cases in a survival analysis violates the basic assumption that all cases are independent. However, several reports have shown that the effect of including bilateral cases in studies of hip and knee joint prosthesis survival is negligible (Robertsson and Ranstam 2003, Lie et al. 2004). We therefore included all available cases to maximize statistical power. Relative risk (RR) estimates were calculated and are presented with 95% CI. The level of significance was 5%.

### Hip resurfacing vs. conventional implant design

The survival rate for HRA was compared to the survival rate of 48,409 THAs performed for similar patients during the same time period, with adjustment for age at surgery, sex, operated side, and diagnosis, using Cox multiple regression (Table 1). In addition, stratified analyses were performed for males and females aged < 55 or ≥ 55 years. In these sub-analyses by age and sex, revision risk for HRA was compared to revision risk for all THAs and separately to revision risk for all cemented THAs performed for similar patients during the same time period.

**Table 1.** Clinical details relating to HRAs and all THAs in 52,810 hips

A	B	C	D	E	F	G	H	I	J
HRA	4,401	3.5 (0–8.7)	55 (9–85)	66	2001–2009	46	11 (1–104)	54	92
All THAs	48,409	3.9 (0–9.0)	68 (14–85)	42	2001–2009	78	69 (1–493)	57	94

A Hip device B n C Mean follow-up (range) D Mean age (range) E % Males F Implanting period G No. of hospitals H Mean volume (range) of operations per hospital per year over the study period I Operated side, % right J Diagnosis, % primary osteoarthritis

### The hip resurfacing group

The HRA group was further analyzed with regard to the influence of age at surgery, sex, operated side, diagnosis, and implant design on the risk of revision. The 6 most commonly used implant designs were studied (ASR, DePuy; BHR, Smith and Nephew; Durom, Zimmer; ReCap, Biomet; Conserve Plus, Wright Medical; and Cormet, Corin Medical) (Table 2), resulting in a study group of 4,401 hips with 159 revisions. Information about femoral head diameter was available in all cases. To investigate correlation with sex, femoral head diameter (classified as ≤ 44 mm, 45–49 mm, 50–54 mm, and ≥55 mm) was added in separate evaluations. This is the same diameter classification as in the Australian Registry and the NARA report (Australian Orthopaedic Association, Johanson et al. 2010).

**Table 2.** HRA implant designs in the Finnish Arthroplasty Register, 2001–2009

Implant design	%	n
BHR	42	1,856
ASR	23	995
ReCap	15	657
Conserve Plus	11	469
Durom	8	333
Cormet	2	91
Total	100	4,401

Revisions were linked to the primary operation by using the patient’s personal identification number. The endpoint for survival was defined as revision when either one component (including the femoral head) or the whole implant was removed or exchanged. Revision for any reason, for aseptic loosening, for dislocation, for infection, and for periprosthetic fracture each served separately as an endpoint. In 13 revisions, the recorded indication for revision was “other reason”. Kaplan-Meier survival data were used to construct the survival probabilities of implants. These survival data were compared using the log-rank test. Patients who died or left Finland during the follow-up period were censored at that point. The Cox multiple regression model was used to study differences between groups and to adjust for potential confounding factors. The factors studied with the Cox model were the 6 hip resurfacing devices, age, sex, diagnosis, and hospital production volume (≥ 100 or < 100 procedures). Effect of age on survivorship was also analyzed by dividing the patients into 2 age groups: those under 55 years and those who were 55 years of age or older. Cox regression analyses provided estimates of survival probabilities and adjusted risk ratios for revision. Estimates from the Cox analyses were used to construct adjusted survival curves at mean values of the risk factors. The Wald test was used to calculate p-values for data obtained from the Cox multiple regression analysis. Differences between groups were considered to be statistically significant if the p-values were less than 0.05 in a two-tailed test. The statistical analyses were conducted with PASW Statistics version 18.

## Results

### Demographics and revisions

66% of the patients were male in the HRA group and 42% were male in the THA group. Mean age of HRA cases was 55 (9–85) years and mean age of THA cases was 68 (14–85) years. Primary osteoarthritis was slightly more common in the THA group (94%) than in the HRA group (92%) (Table 1). The HRA designs in the database are listed in Table 2. The main reason for revision of hip resurfacings was aseptic loosening of both components, whereas THAs were revised mainly because of dislocation. Unspecified reasons for revision (“other”) were recorded in 8% of the HRA revisions as compared to 5% of the THA revisions (Table 3). The 4-year unadjusted Kaplan-Meier survival was 96% (95% CI: 96–97) for both HRA and THA groups (Table 4).

**Table 3.** Reasons for revision

A	B	C	D	E	F	G	H	I	J	K	L
HRA	4,401	41 (26%)	26 (16%)	9 (6%)	10 (6%)	3 (2%)	24 (15%)	31 (19%)	2 (1%)	13 (8%)	159
THA	48,409	339 (19%)	195 (11%)	105 (6%)	199 (11%)	467 (27%)	133 (8%)	204 (12%)	20 (1%)	88 (5%)	1,750
Total	52,810	380 (20%)	221 (12%)	114 (6%)	209 (11%)	470 (25%)	157 (8%)	235 (12%)	22 (1%)	101 (5%)	1,909

A Hip device B n C Aseptic loosening of both components D Aseptic loosening of the cup E Aseptic loosening of the stem F Infection G Dislocation H Malposition I Fracture J Implant breakage K Other reason including local periprosthetic reactions such as metallosis associated with the metal-on-metal articulation. L All

**Table 4.** Survival of HRA and THA, the reference group. Endpoint was defined as revision of any component for any reason. Survival rates were obtained from the Kaplan-Meier analysis

	A	B	C	D	E	F	G	H	I	J
BHR	1,856	4.4 (0.0–9.0)	1,253	97 (96–98)	668	96 (95–97)	111	95 (94–97)	0.72 (0.56–0.94)	0.02
ASR	995	3.1 (0.3–5.8)	440	95 (93–97)	39	92 (89–95)	–	–	1.28 (0.95–1.73)	0.1
ReCap	657	2.5 (0.4–5.7)	190	96 (94–98)	18	–	–	–	0.94 (0.59–1.48)	0.8
Conserve Plus	469	2.1 (0.4–4.7)	65	97 (95–98)	–	–	–	–	1.05 (0.61–1.83)	0.9
Durom	333	3.2 (0.2–5.1)	144	96 (94–98)	1	–	–	–	1.03 (0.60–1.74)	0.9
Corin (Cormet)	91	5.6 (0.2–7.6)	87	96 (92–100)	52	92 (86–99)	5	–	1.11 (0.50–2.48)	0.8
All HRAs	4,401	3.5 (0.0–9.0)	2,177	96 (96–97)	777	94 (93–95)	115	94 (93–95)	0.93 (0.78–1.10)	0.4
All THAs	48,409	3.9 (0.0–9.0)	25,482	96 (96–97)	14,607	95 (95–96)	5,601	94 (94–95)	1.0	–

A n B Mean follow-up (years) C At risk, 4-year D 4-year survival (95% CI) E At risk, 6-year F 6-year survival (95% CI) G At risk, 8-year H 8-year survival (95% CI) I Adjusted risk ratio (RR) for revision (95% CI) from the Cox regression analysis (HRAs compared to THAs; with adjustment made for age, sex, diagnosis, and implant). J p-value

### Hip resurfacing vs. conventional implant designs—overall results

**Figure 1.**
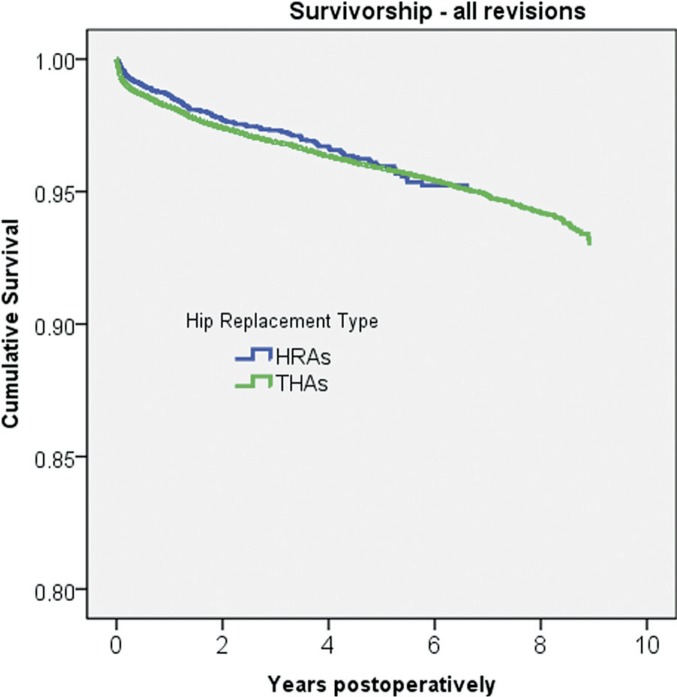
Cox-adjusted survival curves for 4,401 HRAs and 48,409 THAs. The endpoint was defined as revision for any reason. Adjustment was made for age at surgery, sex, operated side, and diagnosis.

There was no statistically significant difference in revision risk between HRAs and THAs (RR = 0.93, CI: 0.78–1.10; p = 0.4) (Table 4, Figure 1) or between HRAs and all-cemented THAs (RR = 0.88, CI: 0.69–1.13; p = 0.3) (Table 5).

**Table 5.** Age- and sex-stratified relative risk of revision. HRAs were compared to all THAs and to all cemented THAs implanted during the same period (2001–2009). Data are based on a Cox regression model adjusted for age, diagnosis, and type of implant

	A	B	C	D
Age ≤ 54 years				
Males	0.83 (0.55–1.23)	0.3	0.55 (0.25–1.20)	0.1
Females	1.05 (0.74–1.49)	0.8	0.96 (0.47–1.95)	0.9
Age ≥ 55 years				
Males	0.74 (0.54–1.01)	0.06	0.73 (0.50–1.08)	0.1
Females	1.38 (0.98–1.94)	0.06	1.36 (0.91–2.02)	0.1

A Adjusted RR^a^ of revision: HRA/all THAs (95% CI) B p-value C Adjusted RR^a^ of revision: HRA/all cemented THAs (95%CI) D p-value ^a^ RR: See Table 4, footnote I.

### Hip resurfacing vs. conventional implant designs—age and sex analysis

There were no statistically significant differences in revision rates between HRAs and THAs or between HRAs and all-cemented THAs in the subgroup analysis, by age and sex (Table 5).

### Hip resurfacing group: the 6 most commonly used designs

**Figure 2.**
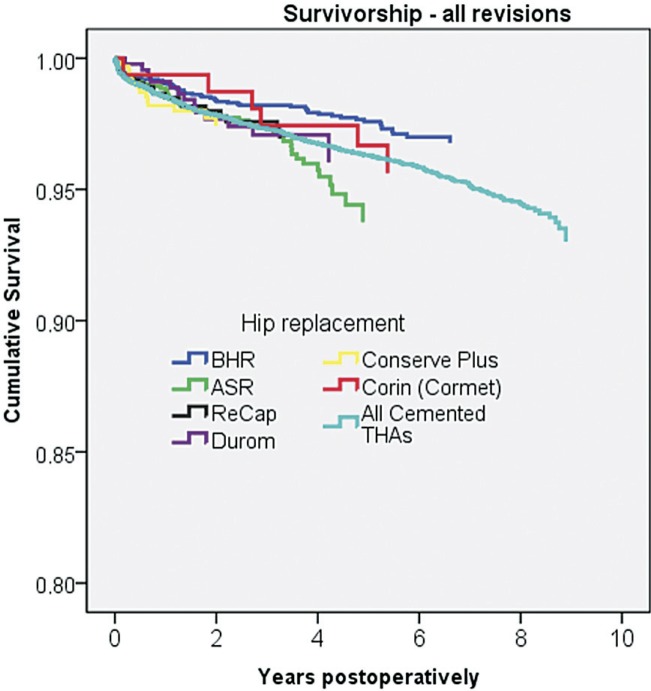
Cox-adjusted survival curves for 6 HRA designs (1,856 BHR, 995 ASR, 657 ReCap, 469 Conserve Plus, 333 Durom, and 91 Corin) and 18,843 cemented THAs. The endpoint was defined as revision for any reason. Adjustment was made for age at surgery, sex, operated side, and diagnosis.

The mean overall follow-up for HRA designs and THAs is presented in Table 4. In Figure 2, the Cox-adjusted survival of HRA designs is compared to that of the cemented THA. Female patients had about twice the revision risk of male patients (RR = 1.98, CI: 1.44–2.71; p < 0.001) (Table 6). In repeated analysis also including the femoral head diameter, gender still had a statistically significant influence on revision rate. However, the femoral head diameter did not influence the revision rate (Table 7). Hospitals that had performed 100 or more HRA procedures had a lower revision risk than those with less than 100 HRAs (RR = 0.61, CI: 0.41–0.88; p = 0.009) (Table 6).

**Table 6.** Relative risk of revision with 95% confidence interval (CI) in 4,401 hip resurfacings (159 revisions). The data are based on a Cox regression model including age (< 54 or ≥ 55 years), sex, diagnosis, and the 6 most common HRA designs with BHR as reference

	RR	95% CI	p-value
BHR (reference)	1	–	
Corin	1.34	0.57–3.16	0.5
Converse Plus	1.60	0.88–2.91	0.1
Durom	1.58	0.86–2.91	0.1
ReCap	1.17	0.68–2.01	0.6
ASR	1.83	1.23–2.72	0.003
Female / Male	1.98	1.44–2.71	< 0.001
Age (< 55 / ≥55 years)	1.05	0.75–1.45	0.8
Secondary / primary OA	1.25	0.75–2.10	0.4
Hospital production volume: ≥ 100 / < 100 procedures	0.61	0.42–0.89	0.009

**Table 7.** The same type of analysis as in Table 6 except that femoral head diameter (categorized as ≤ 44 mm, 45–49 mm, 50–54 mm, and ≥ 55 mm) has been added, resulting in 4,401 resurfacings (159 revisions) available for analysis ^a^

	RR	95% CI	p-value
BHR (reference)	1	–	
ASR	1.73	1.14–2.61	0.01
Female / Male	1.87	1.19–2.92	0.006
Age (< 55 / ≥ 55 years)	1.00	0.72–1.38	1.0
Femoral head diameter			
≤ 44 mm (reference)	1	–	
45–49 mm	0.89	0.41–1.94	0.8
50–54 mm	0.75	0.40–1.41	0.4
≥ 55 mm	0.65	0.38–1.12	0.1

**^a^** Only the results for BHR, ASR, age, sex, and femoral head diameter are given to make the table easier to read.

**Figure 3.**
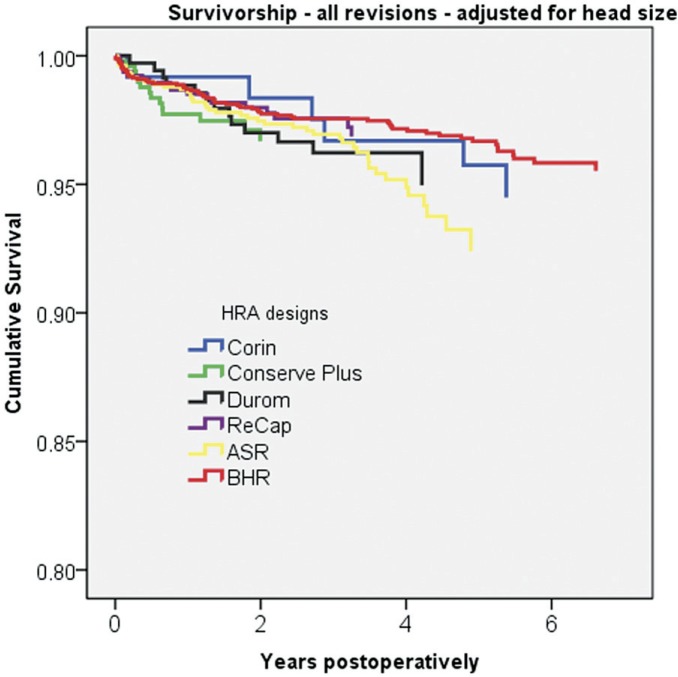
Cox-adjusted survival curves for six HRA designs (1,856 BHR, 995 ASR, 657 ReCap, 469 Conserve Plus, 333 Durom, and 91 Corin). The endpoint was defined as revision for any reason. Adjustment was made for age at surgery, sex, operated side, diagnosis, and femoral head diameter (categorized as ≤ 44 mm, 45–49 mm, 50–54 mm, and ≥ 55 mm).

When we compared the different HRA designs using BHR as a reference, ASR had a higher risk of revision than BHR (RR = 1.83, CI: 1.23–2.72; p = 0.003) (Table 6 and Figure 3). CI for the Durom, ReCap, Converse Plus, and Corin designs showed considerable overlap, and the analysis did not permit any ranking among them.

## Discussion

We found that hip resurfacing arthroplasty had short-term survivorship comparable to that of total hip arthroplasty on a nationwide level. Implant design had an influence on revision rates, as did volume of hospital procedures. Female patients had twice the revision risk of male patients.

We acknowledge that the present study has methodological shortcomings. By conducting a registry-based study, we were not able to compare the functional results between the groups. Nor did we perform any radiological analyses, which could have detected silent osteolysis, neck narrowing (Hing et al. 2007b), or adverse biological reactions linked to metal-on-metal articulation (Shimmin et al. 2005, Keegan et al. 2007, Grammatopolous et al. 2009, Ollivere et al. 2009,). Patients in the HRA group had a lower mean age than patients in the THA group. There were also more male patients in the HRA group. These problems were adjusted for as far as possible by the use of regression models. In theory, of course, selection bias can only be avoided by conducting a randomized controlled trial. However, it has recently been pointed out that well-designed observational studies provide reliable information on treatment effects, and the role of single, randomized controlled studies should not be overemphasized in clinical decisions (Benson and Hartz 2000, Concato et al. 2000).

The follow-up time of 0–9 years was relatively short. The proportion of revisions that could be related to surgical and technical errors was probably high. With longer follow-up, other reasons for revision—and especially those related to wear and toxic reaction caused by wear-related debris or release of metal—might be supposed to change the relative distribution of revisions results (Hing et al. 2007a, Heilpern et al. 2008, Steffen et al. 2008, Khan et al. 2009). Aseptic loosening of the cup was a relatively common finding in our study. Most of these cases are clear clinical entities with failure of osteointegration. These fixation failures are usually diagnosed during the first months after the index operation. However, some patients manage longer periods with moderate symptoms with a loose cup, until the position of the cup finally changes. It is still possible that some adverse reactions to metal debris (ARMDs) are falsely classified as aseptic loosening because of the lack of a specific question about ARMD in the Finnish data collection form. Aseptic loosening of a cementless cup is indeed a short-term complication. In the long term, the osteointegrated cups do not become loose unless there is massive osteolysis caused by, for example, polyethylene debris. ARMD can cause massive soft tissue damage in the hip, but it is seldom the true reason for loosening.

Revisions performed for dislocation are often complex operations. It is true that soft tissue problems seldom exist in the revisions for dislocation, in contrast to the situation with revisions for ARMD. However, most ARMD revisions in Finland have—at least until now—been moderate, and the technical problems in these revisions have been solved using the same options as in other revisions.

The total number of revisions was also relatively low, permitting only a minimum of stratified analysis and increasing the sensitivity to random effects of single revision cases. Most of the good short-term results for hip resurfacing arthroplasty have been published from pioneering centers (Amstutz et al. 2004, Daniel et al. 2004, Treacy et al. 2005). Recent independent reports have confirmed these results (Hing et al. 2007a, Heilpern et al. 2008, Steffen et al. 2008, Khan et al. 2009). However, reports from national joint replacement registries have shown higher revision rates for hip surfacing than for conventional arthroplasty (Johanson et al. 2010, National Joint Registry for England and Wales (NJR England-Wales)). According to the Australian Arthroplasty Register, the cumulative revision rate of HRAs for primary osteoarthritis in 9 years was 7.2% (95% CI: 6.2–8.4) (Australian Orthopaedic Association). In our study, the 8-year survival for HRA was 94% (93–95), which is similar to that published from Australia. The adjusted risk ratio for revision, between HRA and THA, was similar in our study. In the subgroup analyses by age and sex, there were no differences either between HRAs and cemented THAs. However, we found some support for the previous finding that especially elderly women with HRA have an increased risk of revision compared to those with conventional implants (Johanson et al. 2010). In the Nordic Arthroplasty Register Association (NARA) database, there was also a higher revision risk in younger women with HRA than in those with cemented THA (Johanson et al. 2010). The results of cemented implants in young patients in Finland have not been as good as in other Nordic countries (Mäkelä et al. 2011). On the other hand, the number of HRAs implanted in Finland is more than twice as high as that of HRAs implanted in the 3 other Nordic countries together. It is obvious that the amount of HRAs per surgeon has been higher in Finland than in other Nordic countries, which may partly explain the differences in results between previous studies and the current study. Furthermore, in our analysis, patients with aseptic necrosis were excluded. This may have had a positive affect on our results. However, it is also possible that the survival of the Finnish THA is poorer than those in Australia and in Scandinavia due to common usage of cementless implants and wear problems. The HRA survival data may look good when comparing them with those of poor THAs.

The Australian, British, and Scandinavian registries have reported approximately twice the revision rate for women than for men (Australian Orthopaedic Association, National Joint Registry for England and Wales (NJR England-Wales), Johanson et al. 2010). However, based on data from the Australian register, it has been stated that on adjusting for femoral head size, female sex no longer remained as an independent risk factor (Prosser et al. 2010). On the other hand, the NARA group found that femoral head diameter alone had no influence on early revision rate (Johanson et al. 2010). In our analysis of HRA, we also found that the revision rate for females was twice that for men. We could not find any reduced revision risk with increased femoral head diameter, which supports the results of the NARA group.

We used a limit of 100 HRAs to separate low-volume hospitals from high-volume hospitals. Information on operative volume per surgeon was not available. High hospital production volume was associated with a reduced risk of revision. It seems reasonable that in Finland, hip resurfacings should be centralized to high-volume hospitals. HRAs had been performed in 46 hospitals during the study period.

6 designs were analyzed separately. The BHR device was the only implant that had a reduced risk of revision compared to THAs. The THAs included were the most commonly used brands during the study period in Finland. It is possible that some THA implants analyzed separately would have had higher survival than BHR. However, we wanted to compare BHR, the market leader, with the average THA. When the revision risk of other HRA models was compared to that of BHR, there were no statistically significant differences between implants—except for ASR, which had an increased risk of revision. These findings are consistent with results from the Australian, the British, and the Scandinavian registries. 3 of the designs analyzed, ReCap, Conserve Plus and Durom, had a considerably shorter follow-up than BHR. Almost all of the ASRs in Finland were performed in the Coxa Hospital in Tampere, which is one of the largest hip arthroplasty centers in Europe. Although ASRs were implanted by high-volume surgeons, the outcome was poor due to poor implant design. It has been stated that ASR is one of the biggest disasters in orthopedic history (Cohen 2011).

The most common reason for HRA revision in Australia was fracture (36%) followed by loosening/lysis (33%) (Australian Orthopaedic Association). Both femoral and acetabular components were revised in 37% of all revisions (Australian Orthopaedic Association). The most common reason for HRA revision in Scandinavia also was fracture (40%) followed by other reasons (30%) and aseptic loosening (25%) (Johanson et al. 2010). However, the type of revision was not specified in the NARA report. In the present study, the most common reason for HRA revision was aseptic loosening of both components (26% of all cases; 41 of 159 revisions). Moreover, there were 26 cases of aseptic loosening of the cup only (16%) and 9 cases of aseptic loosening of the stem only (6%). Altogether, 76 of 159 HRA revisions (48%) in the Finnish register were performed for aseptic loosening. In clinical practice, one usually has to revise both components of the HRA because of bearing surface compatibility when the HRA cup is revised for early instability. Most of the early problems with aseptic loosening in Finland are therefore probably due to unstable cups because of smooth coating (Durom) and difficult operative techniques. Isolated loose femoral components without fracture were rare in the short term. In our analysis, patients with aseptic necrosis were excluded. This may have had a positive affect on our results of revisions performed for fractures.

There were 3 revisions due to dislocation in the HRA group (0.07% of all HRAs) and 467 in the THA group (1.0% of all THAs). The relatively high dislocation rate of THA should not be forgotten when comparing different devices. Femoral neck fracture was the reason for revision in 19% of all revisions, which is less than in previous reports (Australian Orthopaedic Association, Johanson et al. 2010).

A well-known disadvantage of a metal-on-metal articulation is that it releases a large amount of very small metal (CoCr) particles and ions, which may lead to adverse biological reactions, including local soft tissue toxicity, delayed type hypersensitivity reactions, osteolysis, and even risk of carcinogenesis (Keegan et al. 2007, Shimmin et al. 2005). Nowadays, HRA surgeons are familiar with HRA joint hydrops with milky sterile fluid, soft tissue necrosis, and pseudotumors. It is possible that there will be an increasing number of metal-on-metal bearing surface revisions in the future. In the Finnish Arthroplasty Register notification form, bearing surface complications are not asked separately. It is probable that some of these HRA meta-bearing complications are coded in the Finnish register as revisions performed for “other reason”. However, there were only 13 HRA revisions for “other reason”. It may be that during the past few years, surgeons have not yet been as familiar with this metal bearing problem as they are today, and some of them may have described it as loosening or malposition.

In conclusion, we found that HRA had a short-term survivorship comparable to that of THA at a nationwide level. Implant design had an influence on revision rate, as did hospital procedure volume. Female patients had a revision risk that was twice that of male patients.

## References

[CIT0001] Amstutz HC, Beaule PE, Dorey FJ, Le Duff MJ, Campbell PA, Gruen TA (2004). Metal-on-metal hybrid surface arthroplasty: two to six-year follow-up study. J Bone Joint Surg (Am).

[CIT0002] Australian Orthopaedic Association (2010). National Joint Replacement Registry. Annual Report. http://www.dmac.adelaide.edu.au/aoanjrr/documents/aoanjrrreport_2010.pdf.

[CIT0003] Benson K, Hartz AJ (2000). A comparison of observational studies and randomized, controlled trials. N Engl J Med.

[CIT0004] Cohen D (2011). Revision rates for metal on metal hip joints are double that of other materials. BMJ.

[CIT0005] Concato J, Shah N, Horwitz RI (2000). Randomized, controlled trials, observational studies, and the hierarchy of research designs. N Engl J Med.

[CIT0006] Daniel J, Pynsent PB, McMinn DJ (2004). Metal-on-metal resurfacing of the hip in patients under the age of 55 years with osteoarthritis. J Bone Joint Surg (Br).

[CIT0007] Grammatopolous G, Pandit H, Kwon YM, Gundle R, McLardy-Smith P, Beard DJ, Murray DW, Gill HS (2009). Hip resurfacings revised for inflammatory pseudotumour have a poor outcome. J Bone Joint Surg (Br).

[CIT0008] Heilpern GN, Shah NN, Fordyce MJ (2008). Birmingham hip resurfacing arthroplasty: a series of 110 consecutive hips with a minimum five-year clinical and radiological follow-up. J Bone Joint Surg (Br).

[CIT0009] Hing CB, Back DL, Bailey M, Young DA, Dalziel RE, Shimmin AJ (2007a). The results of primary Birmingham hip resurfacings at a mean of five years. An independent prospective review of the first 230 hips. J Bone Joint Surg (Br).

[CIT0010] Hing CB, Young DA, Dalziel RE, Bailey M, Back DL, Shimmin AJ (2007b). Narrowing of the neck in resurfacing arthroplasty of the hip: a radiological study. J Bone Joint Surg (Br).

[CIT0011] Johanson PE, Fenstad AM, Furnes O, Garellick G, Havelin LI, Overgaard S, Pedersen AB, Karrholm J (2010). Inferior outcome after hip resurfacing arthroplasty than after conventional arthroplasty. Evidence from the Nordic Arthroplasty Register Association (NARA) database, 1995 to 2007. Acta Orthop.

[CIT0012] Keegan GM, Learmonth ID, Case CP (2007). Orthopaedic metals and their potential toxicity in the arthroplasty patient: A review of current knowledge and future strategies. J Bone Joint Surg (Br).

[CIT0013] Khan M, Kuiper JH, Edwards D, Robinson E, Richardson JB (2009). Birmingham hip arthroplasty: five to eight years of prospective multicenter results. J Arthroplasty.

[CIT0014] Lie SA, Engesaeter LB, Havelin LI, Gjessing HK, Vollset SE (2004). Dependency issues in survival analyses of 55,782 primary hip replacements from 47,355 patients. Stat Med.

[CIT0015] Makela KT, Eskelinen A, Pulkkinen P, Paavolainen P, Remes V (2011). Results of 3,668 primary total hip replacements for primary osteoarthritis in patients under the age of 55 years. Acta Orthop.

[CIT0016] National Joint Registry for England and Wales (NJR England-Wales) (2010). 7th Annual Report. http://www.njrcentre.org.uk.

[CIT0017] Ollivere B, Darrah C, Barker T, Nolan J, Porteous MJ (2009). Early clinical failure of the Birmingham metal-on-metal hip resurfacing is associated with metallosis and soft-tissue necrosis. J Bone Joint Surg (Br).

[CIT0018] Paavolainen P, Hamalainen M, Mustonen H, Slatis P (1991). Registration of arthroplasties in Finland. A nationwide prospective project. Acta Orthop Scand.

[CIT0019] Prosser GH, Yates PJ, Wood DJ, Graves SE, de Steiger RN, Miller LN (2010). Outcome of primary resurfacing hip replacement: evaluation of risk factors for early revision. Acta Orthop.

[CIT0020] Puolakka TJ, Pajamaki KJ, Halonen PJ, Pulkkinen PO, Paavolainen P, Nevalainen JK (2001). The Finnish Arthroplasty Register: report of the hip register. Acta Orthop Scand.

[CIT0021] Robertsson O, Ranstam J (2003). No bias of ignored bilaterality when analysing the revision risk of knee prostheses: analysis of a population based sample of 44,590 patients with 55,298 knee prostheses from the national Swedish Knee Arthroplasty Register. BMC Musculoskelet Disord.

[CIT0022] Shimmin AJ, Bare J, Back DL (2005). Complications associated with hip resurfacing arthroplasty. Orthop Clin North Am.

[CIT0023] Sibanda N, Copley LP, Lewsey JD, Borroff M, Gregg P, MacGregor AJ, Pickford M, Porter M, Tucker K, van der Meulen JH (2008). Revision rates after primary hip and knee replacement in England between 2003 and 2006. PLoS Med.

[CIT0024] Steffen RT, Pandit HP, Palan J, Beard DJ, Gundle R, McLardy-Smith P, Murray DW, Gill HS (2008). The five-year results of the Birmingham Hip Resurfacing arthroplasty: an independent series. J Bone Joint Surg (Br).

[CIT0025] Treacy RB, McBryde CW, Pynsent PB (2005). Birmingham hip resurfacing arthroplasty. A minimum follow-up of five years. J Bone Joint Surg (Br).

